# Sexual Dimorphism in the Closure of the Hippocampal Postnatal Critical Period of Synaptic Plasticity after Intrauterine Growth Restriction: Link to Oligodendrocyte and Glial Dysregulation

**DOI:** 10.1159/000530451

**Published:** 2023-04-05

**Authors:** Michael Nugent, Mark St. Pierre, Ashley Brown, Salma Nassar, Pritika Parmar, Yuma Kitase, Sarah Ann Duck, Charles Pinto, Lauren Jantzie, Camille Fung, Raul Chavez-Valdez

**Affiliations:** a Division of Neonatal-Perinatal Medicine, Department of Pediatrics, Johns Hopkins School of Medicine, Baltimore, MD, USA; b Division of Neonatology, Department of Pediatrics, University of Utah, Salt Lake City, UT, USA; c Department of Neurosciences, Johns Hopkins University Krieger School of Arts and Sciences, Baltimore, MD, USA; d Department of Molecular and Cellular Biology, Johns Hopkins University Krieger School of Arts and Sciences, Baltimore, MD, USA; e Department of Human Biology, University of Toronto, Toronto, ON, Canada

**Keywords:** Microglia, Cytokines, Intrauterine growth restriction, Memory, Neuropsychiatric disorders, Synaptic plasticity

## Abstract

Intrauterine growth restriction (IUGR) resulting from hypertensive disease of pregnancy (HDP) leads to sexually dimorphic hippocampal-dependent cognitive and memory impairment in humans. In our translationally relevant mouse model of IUGR incited by HDP, we have previously shown that the synaptic development in the dorsal hippocampus including GABAergic development, NPTX2^+^ excitatory synaptic formation, axonal myelination, and perineural net (PNN) formation were perturbed by IUGR at adolescent equivalence in humans (P40). The persistence of these disturbances through early adulthood and the potential upstream mechanisms are currently unknown. Thus, we hypothesized that NPTX2^+^ expression, PNN formation, axonal myelination, all events closing synaptic development in the hippocampus, will be persistently perturbed, particularly affecting IUGR female mice through P60 given the fact that they had worse short-term recognition memory in this model. We additionally hypothesized that such sexual dimorphism is linked to persistent glial dysregulation. We induced IUGR by a micro-osmotic pump infusion of a potent vasoconstrictor U-46619, a thromboxane A_2_-analog, in the last week of the C57BL/6 mouse gestation to precipitate HDP. Sham-operated mice were used as controls. At P60, we assessed hippocampal and hemispheric volumes, NPTX2 expression, PNN formation, as well as myelin basic protein (MBP), Olig2, APC/CC1, and M-NF expression. We also evaluated P60 astrocytic (GFAP) reactivity and microglial (Iba1 and TMEM119) activation using immunofluorescent-immunohistochemistry and Imaris morphological analysis plus cytokine profiling using Meso Scale Discovery platform. IUGR offspring continued to have smaller hippocampal volumes at P60 not related to changes in hemisphere volume. NPTX2^+^ puncta counts and volumes were decreased in IUGR hippocampal CA subregions of female mice compared to sex-matched shams. Intriguingly, NPTX2^+^ counts and volumes were concurrently increased in the dentate gyrus (DG) subregion. PNN volumes were smaller in CA1 and CA3 of IUGR female mice along with PNN intensity in CA3 but they had larger volumes in the CA3 of IUGR male mice. The myelinated axon (MBP^+^) areas, volumes, and lengths were all decreased in the CA1 of IUGR female mice compared to sex-matched shams, which correlated with a decrease in Olig2 nuclear expression. No decrease in the number of APC/CC1^+^ mature oligodendrocytes was identified. We noted an increase in M-NF expression in the mossy fibers connecting DG to CA3 only in IUGR female mice. Reactive astrocytes denoted by GFAP areas, volumes, lengths, and numbers of branching were increased in IUGR female CA1 but not in IUGR male CA3 compared to sex-matched shams. Lastly, activated microglia were only detected in IUGR female CA1 and CA3 subregions. We detected no difference in the cytokine profile between sham and IUGR adult mice of either sex. Collectively, our data support a sexually dimorphic impaired closure of postnatal critical period of synaptic plasticity in the hippocampus of young adult IUGR mice with greater effects on females. A potential mechanism supporting such dimorphism may include oligodendrocyte dysfunction in IUGR females limiting myelination, allowing axonal overgrowth followed by a reactive glial-mediated synaptic pruning.

## Introduction

The “Barker hypothesis” of fetal origins of disease proposes that early undernutrition leads to permanent changes in structure, metabolism, and function of developing organs [1]. Fetal undernutrition results in intrauterine growth restriction (IUGR), defined as the fetus’ inability to attain predicted growth based on genetic potential [2]. Hypertensive disease of pregnancy (HDP) resulting in uteroplacental insufficiency and fetal malnutrition is the most common cause of IUGR in the developed world [3]. IUGR increases brain-related morbidities [4–6] such as decreasing regional connectivity [7], producing hippocampal hypoplasia [8–11], and resulting in impaired cognition [12, 13] and memory [8, 9]. These human findings are well replicated in multiple animal models of IUGR [14–17].

During the postnatal critical period of synaptic plasticity (pCP), profound remodeling of brain circuits occurs, led by axonal outgrowth, synaptic formation, and pruning to create mature networks shaped by experiences [18]. We have recently reported that HDP-induced IUGR leads to alterations in many of the events governing the boundaries of this pCP in the hippocampus [19]. Decreased polysialylation of the neural cell adhesion molecule in the excitatory neuronal membranes and premature GABAergic network development [18, 20–24] accelerate the opening of the pCP in the dorsal hippocampus [19]. Decreased neuronal pentraxin 2 (NPTX2 or Narp) suggests decreased excitatory input onto parvalbumin (PV)^+^ interneurons [25, 26], which along with impaired perineural net (PNN) formation and myelination suggest disrupted progression and closure of the pCP in IUGR offspring up to P40 (human equivalent to adolescence) [18, 19, 27–31]. Dysregulated temporal progression of the pCP in the hippocampus, which typically closes during late adolescence (>P30) in rodents [23, 32–35], may result in alterations in the excitatory/inhibitory balance and neurobehavioral deficits. The persistence of these perturbations through early adulthood and the potential upstream mechanisms remain unknown.

Early neuroinflammation is regarded as one of the most studied mechanisms to explain the long-lasting deficits linked to IUGR [36, 37]. Intrauterine release of placental damage-associated molecular patterns and direct brain toxicity secondary to chronic hypoxia and hypoperfusion are the two main mechanisms by which neuroinflammation proceeds [38, 39]. Postnatal circulating cytokines increase during the first 2 weeks of life in human IUGR neonates, suggesting an ongoing pro-inflammatory state [40, 41]. Of the most prominent events governing the boundaries of the pCP, disruption of myelination has been most consistently linked to neuroinflammation during early development [42]. Whether astrocytic reactivity and microglia activation lead to neuroinflammation in early adulthood in IUGR offspring is unknown.

Here, using a mouse model physiologically relevant to human HDP-induced uteroplacental insufficiency, we studied the effects of IUGR on the molecular and structural events closing the pCP in the hippocampus, which is critically linked to memory processes [43]. We hypothesized that early adult (P60) NPTX2^+^ expression, PNN formation, axon myelination, all events closing the synaptic development in the hippocampus, will be persistently disturbed in IUGR, particularly affecting IUGR female mice linked to oligodendrocyte and glial dysregulation.

## Materials and Methods

### Animals

**Fig. 1.**
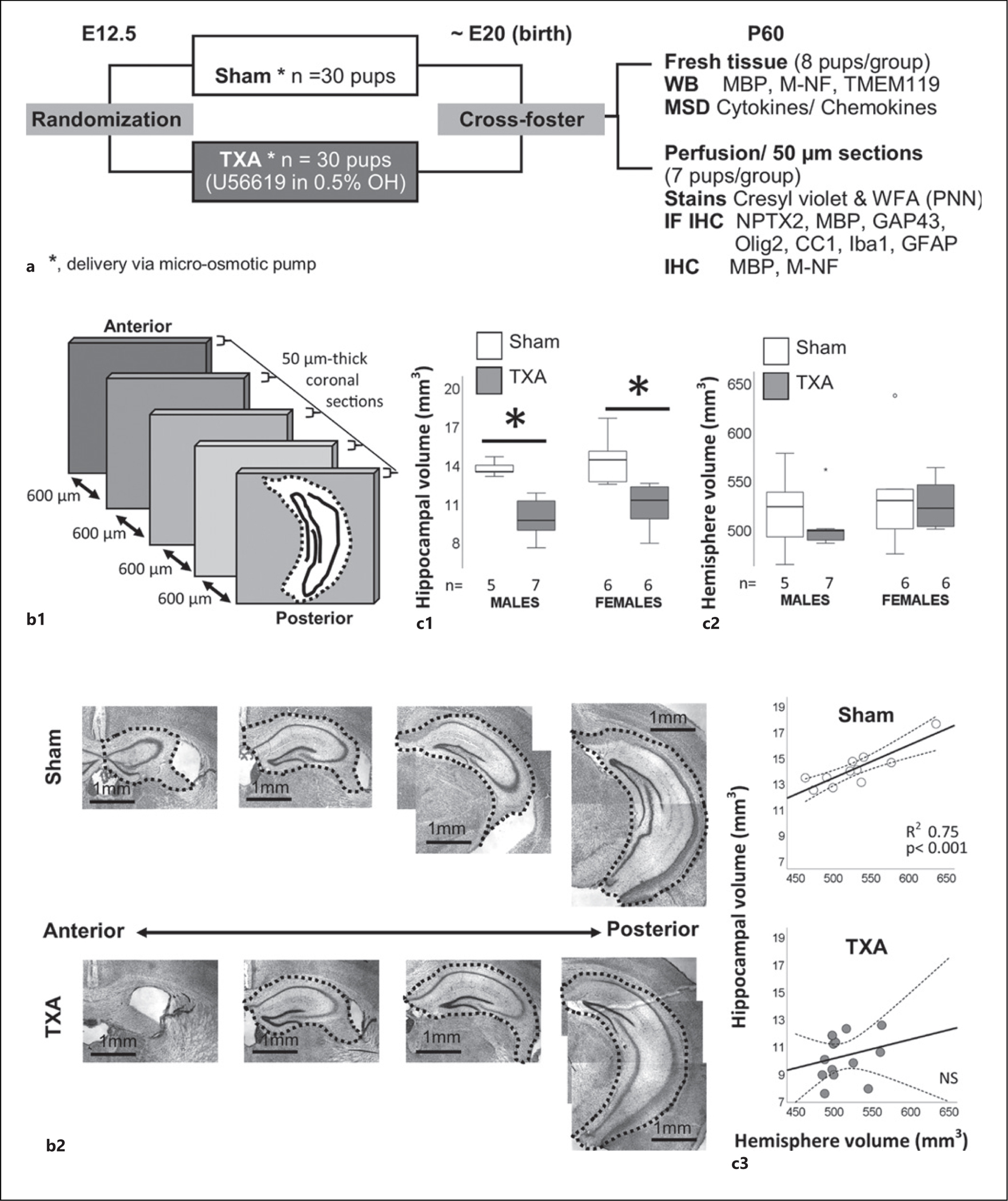
Experimental design and semi-quantitative assessment of hippocampal and hemisphere volume. **a** Pregnant dams were randomized to micro-osmotic pumps containing either vehicle (0.5% ethanol, OH) or U56619 in 0.5% ethanol (TXA) implanted at E12.5. Pups were born at E20 and were cross-fostered to unmanipulated dams. For these experiments, mice of both sexes survived to P60. **b1**, **b2** Perfused brains were sectioned in coronal plane at 50 μm, and every 13th section (600 μm apart) was mounted for cresyl violet staining. Hippocampal areas in all sections were measured and used to extrapolate a semi-quantitative hippocampal volume as previously published [44–46]. Similar methods were used for semi-quantitative hemisphere volume calculation. Box and whisker plots showing median and IQR (25–75% tile) are used to represent the calculated hippocampal volume (**c1**) and hemisphere volume (**c2**) (*p ≤ 0.05 vs. sham, *n* = 5–7 per group). **c3** Scatter plots represent the relationship between hemisphere volume (mm^3^, *x*-axis) and hippocampal volume (mm^3^, *y*-axis) in sham (top) and TXA (bottom) groups, with best fitted regression line (continuous line) with the 95% tile confidence interval (discontinuous lines) shown. R2 and *p* value derived from linear regression are shown. E, embryonic day; MBP, myelin basic protein; M-NF, medium chain neurofilament; MSD, Meso Scale Discovery; NPTX2, neuronal pentraxin 2; Olig2, oligodendrocyte transcription factor; PNN, perineural nets; TXA, thromboxane A_2_-analog; WFA, Wisteria floribunda lectin; IQR, interquartile range.

The University of Utah Animal Care Committee (19-02001) approved all animal procedures in accordance with the NIH Guide for the Care and Use of Laboratory Animals. Details on the model have been published previously [15, 17, 19]. Briefly, at embryonic day (E) 12.5, pregnant C57BL/6J mice (000664, The Jackson Laboratory, Bar Harbor, ME) were anesthetized and a micro-osmotic pump (1007D, 0.5 μL/h, Durect Corporation, Cupertino, CA) infusing either 0.5% ethanol (vehicle, sham) or 4,000 ng/μL of U-46619, a thromboxane A_2_-analog (TXA, Cayman Chemical, Ann Arbor, MI) dissolved in 0.5% ethanol, was retroperitoneally implanted over the last week of gestation. After delivery at ~E20, pups were cross-fostered to unmanipulated dams to minimize complications resulting from surgery (Fig. 1a). One male and one female pup from each litter of sham or IUGR group were used per experiment (fresh tissue or perfusion); therefore, we used 8 dams/treatment group (sham or TXA) = (8 × 2) = 16 pregnant dams in total (with 100% survival rate), and 32 pups (16 males and 16 females) were used for fresh tissue and 28 pups (7 males and 7 females) were used for perfusion. In total, 60 pups were used for all experiments described here. Dams receiving U-46619 developed maternal hypertension by 24 h after pump implantation, and offspring were in average 15% smaller at birth compared to sham [15]. In our hands, weight deficits in IUGR pups worsen until P18 and attenuated by P40 (online suppl. Fig. 1; for all online suppl. material, see www.karger.com/doi/10.1159/000530451), with no significant weight differences identified at P60.

### Brain Harvesting and Tissue Processing

Since we have demonstrated sexual dimorphism in many of the events closing the pCP of synaptic plasticity in the hippocampus by adolescent equivalence (P40) [19], we targeted early adulthood (P60) here, a time at which the events closing the pCP should have concluded in the rodent hippocampus [23, 32–35]. Mice were anesthetized using ketamine (80–100 μg/g of body weight [BW]) and xylazine (7.5–16 μg/g of BW). Brains were harvested after perfusion and fixation for sectioning and immunohistochemistry or via microdissection of the hippocampi for Meso Scale Discovery platform and Western blot analysis.

#### Perfusion/Fixation

Transcardiac infusion of 0.9% normal saline (0.9%) was followed by 4% paraformaldehyde perfusion for fixation. After brain removal, post-fixation proceeded by immersion in 4% paraformaldehyde for 2 h at room temperature (RT), as previously reported [44]. Brains were cryoprotected using a 5%, 10%, 20% sucrose gradient. Brains were transferred in 20% sucrose and shipped on ice overnight to Johns Hopkins University – School of Medicine Laboratory of Neonatology (RCV laboratory) for further processing. At arrival, brains were snap-frozen in dry-ice cold 2-methylbutane, stored at −80°C, and then sectioned in coronal plane using a freezing microtome.

#### Fresh Tissue

For those mice assigned to fresh tissue, decapitation occurred under anesthesia, followed by removal of the skull, isolation of the cerebellum and diencephalon, to finally expose and microdissect the hippocampus in a freezing plate and snap-frozen in dry-ice cold 2-methylbutane and stored at −80°C. Both sexes were equally represented in all groups, and stratification was performed to look for sexual dimorphism.

### Western Blotting

Brain homogenates from naive mice were used to validate antibodies. Homogenates were cryoprotected using 20% (w/v) glycerol and the Bradford assay was used to determine protein concentrations [47] in a 3:1 (v:v) dilution of 35 μg of protein homogenate and 4X loading buffer for Western blots under reducing conditions. Specimens were run in 4–20% mini-protean TGX polyacrylamide precast protein gels (Bio-Rad Inc., Hercules, CA) at 200 V and then transferred to nitrocellulose membrane using Trans-Blot Turbo Mini size (Bio-Rad Inc.). Ponceau S staining was used to verify successful protein transfer and loading. Membranes were blocked with 2% normal goat serum (NGS) in 0.1% Tween/TBS (TBS-T) for validation of antibodies to replicate immunohistochemistry (IHC) experimental conditions and with 2% bovine albumin (BSA) for standard Western blot [44]. Membranes were incubated with primary antibodies overnight at 4°C. After washes, membranes were exposed to secondary antibodies for 1 h and development using enhanced chemiluminescence (Clarity Western ECL Substrate, Bio-Rad Inc.). Validation of antibody criteria included (i) identifiable band(s), at molecular weight(s) described for the target proteins and (ii) absent of nonspecific binding at molecular weights not reported for the protein, except for well-reported post-transcriptional modifications also detected by the antibody. Ponceau S optical density (Fiji/Image J software, NIH) was used for loading control as it incorporates efficiency of protein transfer to the nitrocellulose paper to improve the accuracy of the relative quantification of target proteins. Antibodies for Western blot: (1) neurofilament medium chain subunit (M-NF, EMD Millipore, Billerica MA, USA, AB1987; RRID: AB_91201) rabbit polyclonal IgG antibody raised against recombinant fusion protein containing the C-terminal 168 amino acids of rat NF-M (1 μg/mL, 1:1,000) and (2) TMEM119 (Thermo Fisher Scientific, Waltham MA, USA, PA5–119902; RRID: AB_2913474) rabbit polyclonal IgG antibody raised against a synthetic peptide within human TMEM119 aa 11–52/283 (1 μg/mL, 1:1,000).

### Standard DAB IHC and Nissl Staining

Using a freezing microtome, we produced 50 μm-thick sections to perform IHC for myelin basic protein (MBP) and M-NF as previously described [48]. Primary antibodies include 1) MBP (BioLegend, San Diego CA, USA, 808401; RRID: AB_2564741) mouse monoclonal IgG2b antibody (0.5 μg/mL, 1:2,000) and 2) M-NF (EMD Millipore, Billerica MA, USA, AB1987; RRID: AB_91201) rabbit polyclonal IgG antibody raised against recombinant fusion protein containing the C-terminal 168 amino acids of rat NF-M (2.5 μg/mL, 1:400) followed by goat anti-rabbit antibody (1:200) secondary antibody and DAB as a chromagen. Nissl staining was used for semi-quantitative volumetric analysis. We have reported the hippocampal hypoplasia in this model previously up to P40 [17].

### Semi-Quantitative Assessment of Hippocampal and Hemispheric Volumes

Details have been provided elsewhere for assessment of the hippocampus [44–46]. Brains were sectioned in the freezing microtome at 50 μm in the antero-posterior axis, and every 13th section (600 μm apart) was mounted for Nissl staining. On average, 10–11 sections were obtained per brain and used for hemispheric volume calculations. All slices in which the hippocampus was embedded (on average 5 sections, Fig. 1b1, b2) were used for calculation of hippocampal volumes. The cerebellum was not included in the hemisphere calculation. The areas of hemispheres or hippocampi were averaged and used to semi-quantitatively calculate volumes using the following formula:

(1) $$\begin{array}{rr} \text{Calculated}\;\text{volume}\;\left({\text{mm}}^{3}\right)= & \;\sum_{i=1\;\left(\text{n}=1\right)}\left[{\text{S}}_{\text{i}}\times 0.05\;\text{mm}\right]\\ & \;+\sum_{\text{i}=1\;\left(\text{n}=\text{i}-1\right)}\left[\frac{\left({\text{S}}_{\text{i}}\;+\;{\text{S}}_{\text{i}+1}\right)}{2\;\times \;0.6\;\text{mm}}\right] \end{array}$$

S = hemispheric or hippocampal area (mm^2^), i = section position in anterior-posterior plane, *n* = number of sum repeats.

### Immunofluorescent-IHC

Coronal brain sections containing the dorsal hippocampus were identified as previously reported [19]. Following TBS, pH 7.2 washes for 10 min (× 3 times), antigen retrieval with sodium citrate buffer pH 6.0 for 90 min at 80°C was followed by permeabilization with 0.6% Triton X in TBS for 15 min at RT and blocking with 10% NGS in 0.1% TBS-T for 1 h at RT. Sections were exposed to combinations of the following primary antibodies: (1) rabbit polyclonal IgG anti-NPTX2 (ProteinTech Group Inc., Rosemont, IL; 10889-1AP, 1; 400); (2) mouse IgG2b anti myelin basic protein (MBP; BioLegend Inc., San Diego, CA; 808402; 1:2,000); (3) mouse monoclonal IgG2b anti-CC1/APC (GeneTex, Irvine, CA, USA, GTX16794; RRID: AB_422404, 1:200); (4) rabbit polyclonal IgG anti-Olig2 (EMD Millipore, Billerica MA, USA, AB9610; RRID: AB_570666, 1:500); (5) chicken polyclonal IgY anti-GFAP (Novus, Littleton Co., USA, NBP1-05198; AB_1556315, 1:2,000); and (7) rabbit polyclonal IgG anti-Iba1 (FUJIFILM Wako, Osaka, Japan, 019-19741; RRID: AB_839504, 1:400). Primary antibodies diluted in 4% NGS in TBS (only for NPTX2) or TBS-T (the rest) were incubated overnight at 4°C. Two-hour incubation with goat anti-chicken IgY Alexa Fluor 488, goat anti-rabbit or anti-mouse conjugated to either Alexa Fluor 568 or 647, emitting red and deep red fluorescence signal, respectively (Thermo Fisher Scientific, Inc., Waltham, MA), mixed in 4% NGS/TBS-T, was performed at RT. Staining with 4′,6-diamidino-2-phenylindole (DAPI) at 1 μg/mL in TBS was followed by washes, mounting, and coverslipping using ProLong Glass Antifade Mountant (Molecular Probes, Life Technologies Corp., Carlsbad, CA). Non-primary antibody and subtype- and species-specific immunoglobulin replacing primary antibody negative controls were also run as part of validation experiments [44, 46].

### Wisteria Floribunda Lectin Fluorescent Staining

Brain sections (50 μm thick) were washed in 1X PBS for 10 min, blocked in 10% NGS/PBS at RT for 90 min, and incubated in green fluorescein-conjugated Wisteria floribunda lectin (Vector Laboratories, FL-1351-2; at 1:400) in 4% NGS in PBS for 6 h at RT. Slices were stained in 1 μg/mL DAPI solution for 5 min, washed in PBS, mounted and coverslipped with ProLong Glass Antifade Mountant.

### Confocal Microscopy

Images for Z-stacks were captured at 1,440 × 1,440 pixels, 16-bit, and averaged X2, using a Plan-Apochromat 63X/1.4 oil DIC M27 objective and 1.0 zoom to produce 101.54 × 101.54 μm uncompressed images at the most dorsal CA1 (two fields) and the most temporal CA3 (2 fields) in 2 slices 600 μm apart. Z-stacks were set at 1 air unit (0.8 μm slicing) to 639 nm wavelength using a Laser Scanning Confocal Microscope LSM700 AxioObserver (Carl Zeiss AG, Oberkochen, Germany). For MBP staining, fields were taken at 0.5 zoom, which captures and are of 203.08 × 203.08 μm per panel, taken at 0.5 air unist (0.3 μm). Protocol specifications were followed in all repeats of each experiment; thus, pinhole, gain, and offset configurations were reused from previous experiments. Detection wavelengths were 300–483 nm for DAPI, 493–550 nm for Alexa 488, 560–600 nm for Alexa 568, 644–800 nm for Alexa 647. Uncompressed czi format was used for processing.

### Image Processing Using Imaris

Quantitative analysis was performed using Imaris x64 v9.8.0 software (Bitplane AG, Belfast, UK). We collected 3 primary measurements per immunofluorescent channel: (i) counts of cell, nuclei, or puncta; (ii) per count and total (sum) volumes (μm^3^); and (iii) total intensity of immunofluorescence (in arbitrary units [AU]). Additional measurements included length (μm), area (μm^2^), and number of Sholl intersections (per 1 μm Sholl sphere resolution) for filament analysis (MBP, GFAP, and Iba1). Measurements reported were adjusted for the field volume (μm^3^) of region of interest within the z-stack in which they were imaged.

#### Surface Reconstruction

Following creation of an algorithm that defines channel source and level of detail, the detected objects were automatically thresholded after background subtraction or masking interfering surfaces to zero voxels. Split touching objects function was enabled with the seed point diameter (μm) set to the average diameter of the cells/objects of interest.

#### Filament Reconstruction for MBP, GFAP, and Iba1

An automatic filament detection protocol was used for conjugated GFAP+ (green channel, Alexa 488) and batched for conjugated MBP (deep red, Alexa 647). Creation parameters involved starting point threshold range covering 10% of the upper third quartile for even distribution throughout the field. Seed points were adjusted using automatic detection; these were then manually decreased equilaterally (2 × 104) and seed points were removed around a set diameter of the starting point region to mitigate false filament creation, excess branching, and “spider-webbing.” For conjugated Iba1+ cells (red channel, Alexa 568), we created new filaments with the autopath algorithm setting. Starting point diameter (largest process diameter, placed in the soma or domain center) and seed points’ size (smallest diameter) were identified in slice mode. For creation, starting points were identified semi-manually by thresholding to ~90% coverage and individually adding the remaining points; accuracy was checked at this step as well. The aforementioned seed point creation parameters were utilized. Detected points were then automatically connected with lines following the path of image intensity, with the diameter of these lines being calculated from the fluorescence as well. Any creation abnormalities were corrected by editing segments, branches, or starting points of filaments. Vantage plot was used to consolidate and export detailed statistics outlined above.

### Cytokine/Chemokine Multiplex Analysis

Cytokines and chemokine concentrations were measured in hippocampal crudes using the V-plex multiplex electrochemiluminescent immunoassay platform (Meso Scale Discovery, Gaithersburg, MD, United States) as previously reported by us [49]. This highly sensitive platform has been validated and has inter-assay variations less than 12%. Specifically, tissue lysate (150 μg) or standard was loaded on to a 96-well plate in duplicate per manufacturer’s specification. Plates were read on a QuickPlex SQ 120 Imager (Meso Scale Discovery, Gaithersburg, MD, USA).

### Statistics

After Shapiro-Wilk test confirmed non-normal distribution of the data, non-parametric Mann-Whitney U test was applied stratified by sex since the interest is not in how sex modulates the effect of IUGR as much as how IUGR behaves in each sex. Results were for the most part presented as box and whisker plots, where each box was limited by the 25th and 75th percentiles and the solid line represented the median with all experimental data points shown. Outliers beyond 1.5 × interquartile range from median and extremes beyond 3 × interquartile range from median are represented. The exception was the morphometric measurements for astrocytes and microglia, which were presented as distribution bar graphs. Spearman Rho correlation was used to evaluate the relationship between hemispheric and hippocampal volumes and MBP length and Olig2 expression, and the best fitted regression line with 95th confidence interval was represented against a scatter plot by sex. Lastly, the distribution of Sholl intersections as they distanced from the soma was analyzed using Kolmogorov-Smirnov test. Significance was assigned by *p* value ≤0.05 in all cases. Analysis was performed using IBM SPSS Statistics 28v (IBM Corporation, Armonk, NY).

## Results

### Hippocampal Hypoplasia Persists through Early Adulthood in IUGR Offspring

In our HDP-induced IUGR model, hippocampal hypoplasia was evident at P18 and P40 with IUGR females having the smallest volumes [17]. Using the same methodology, we found that the hippocampal volumes of IUGR mice at P60 were 22.6% (MW U (3) *p* = 0.015 vs. sham) and 26.9% (MW U (1) *p* = 0.004 vs. sham) smaller in males and females, respectively, compared to sex-matched shams (Fig. 1c1). In contrast, hemispheric volume was similar between sham and IUGR mice of either sex (Fig. 1c2). Thus, hemispheric volume predicted hippocampal volume in sham mice (*R*^2^: 0.75, *p* < 0.001) but not in IUGR mice (Fig. 1c3).

### Persistent Deficit in NPTX2^+^ Synapses in IUGR Offspring

**Fig. 2.**
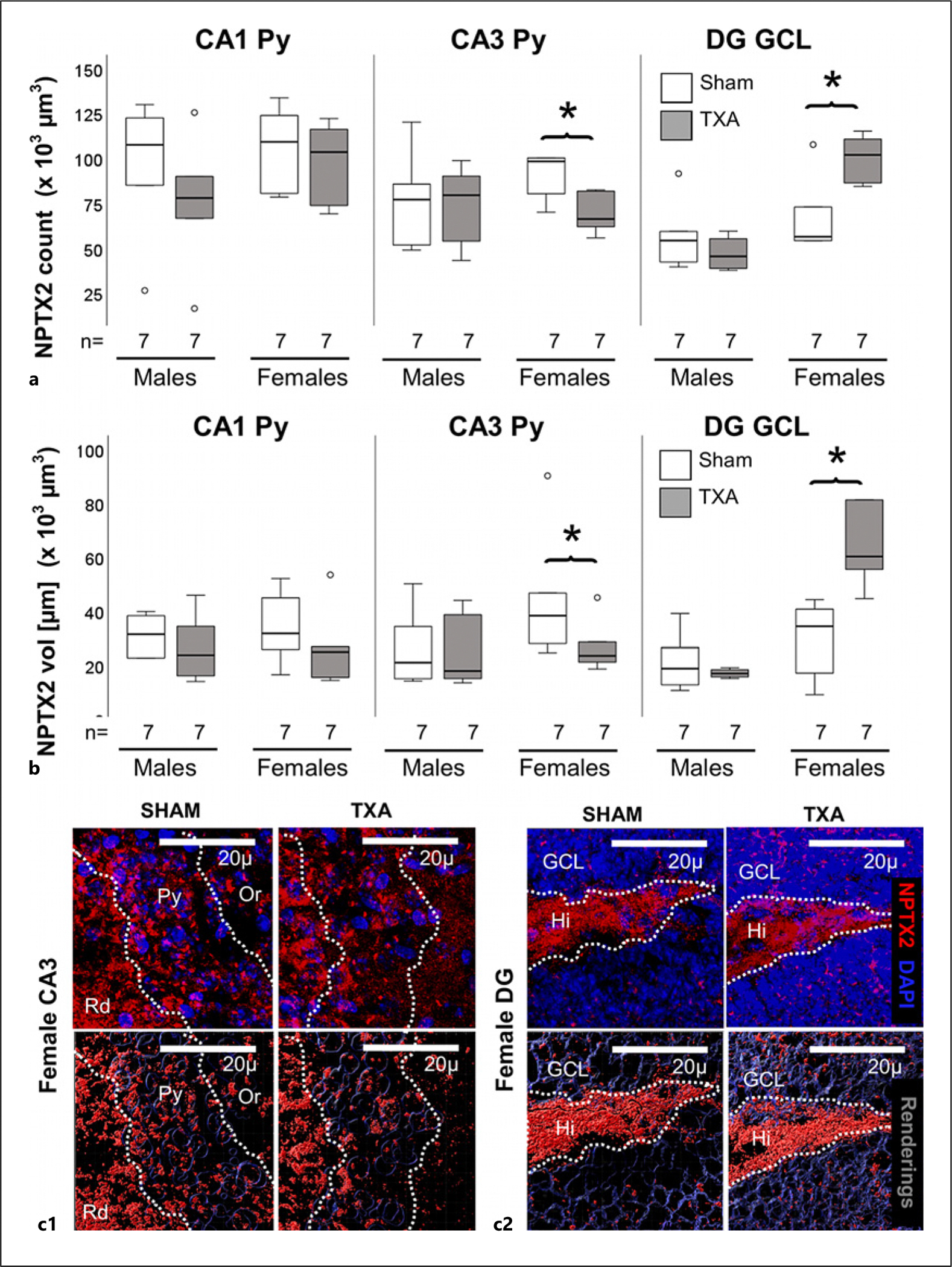
Decreased NPTX2 levels in the CA3 in female IUGR mice at P60. Analysis performed in z-stacks captured from immunolabeling detecting NPTX2 IR in red (Alexa 568 goat anti-rabbit), and DAPI nuclear staining in the dorsal hippocampus. Box and whiskers plots represent NPTX2 number of puncta (per 10^3^ μm^3^, **a**) and total volume (μm^3^ × 10^3^ μm^3^, **b**) at P60 in CA1, CA3, and DG pyramidal cell layer (Py). Boxes are limited by the 75th and 25th percentiles (interquartile range [IQR]) and whiskers are limited by the last data point within 1.5 times the IQR from the median (continuous line inside the box), with outliers represented as °. Mann-Whitney U test was applied. *p < 0.05. Unbiased image processing and analysis was performed using Imaris x64 v9.8 software blinded to treatments and sex. Representative reconstruction renderings from z-stacks using Zen blue software in CA3 (**c1**) and DG (**c2**). CA, cornus ammonis; Or, oriens layer; Py, pyramidal cell layer; Rd, radiatum layer.

Excitatory NPTX2-expressing synapses normally provide regulatory feedback to PV^+^ interneurons. In this model, we previously found that accelerated GABAergic PV^+^ development in the IUGR hippocampal CA subregion at P18 did not result in an increase in NPTX2^+^ synapses, but instead we saw a decrease that was similar between IUGR males and females which resolved by P40 [19]. Because NPTX2 is also necessary for maintenance of PNNs, which are still deficient in female mice at P40 [19], we evaluated if NPTX2 deficit would re-emerge in female mice during early adulthood. Indeed, NPTX2^+^ puncta count and volume (mean rank) decreased by 50% (MW U (5.0) *p* = 0.037 vs. sham) and 47% (MW U (6) *p* = 0.05 vs. sham, *n* = 7/group), respectively, in the CA3 pyramidal cell (Py) layer of IUGR female mice at P60 (Fig. 2a–c). In contrast to the CA3, NPTX2^+^ puncta count and volume (mean rank) were increased by 123% (MW U (22) *p* = 0.047 vs. sham, Fig. 2a) and 166% (MW U (25) *p* = 0.009 vs. sham, Fig. 2b), respectively, in the granular cell layer of the dentate gyrus (DG) of female IUGR mice. No differences were detected in the hilus of the DG. No differences were observed in the hippocampi of male mice.

### Disturbed Closure of the pCP of Synaptic Plasticity in the Hippocampus of IUGR Female Mice

**Fig. 3.**
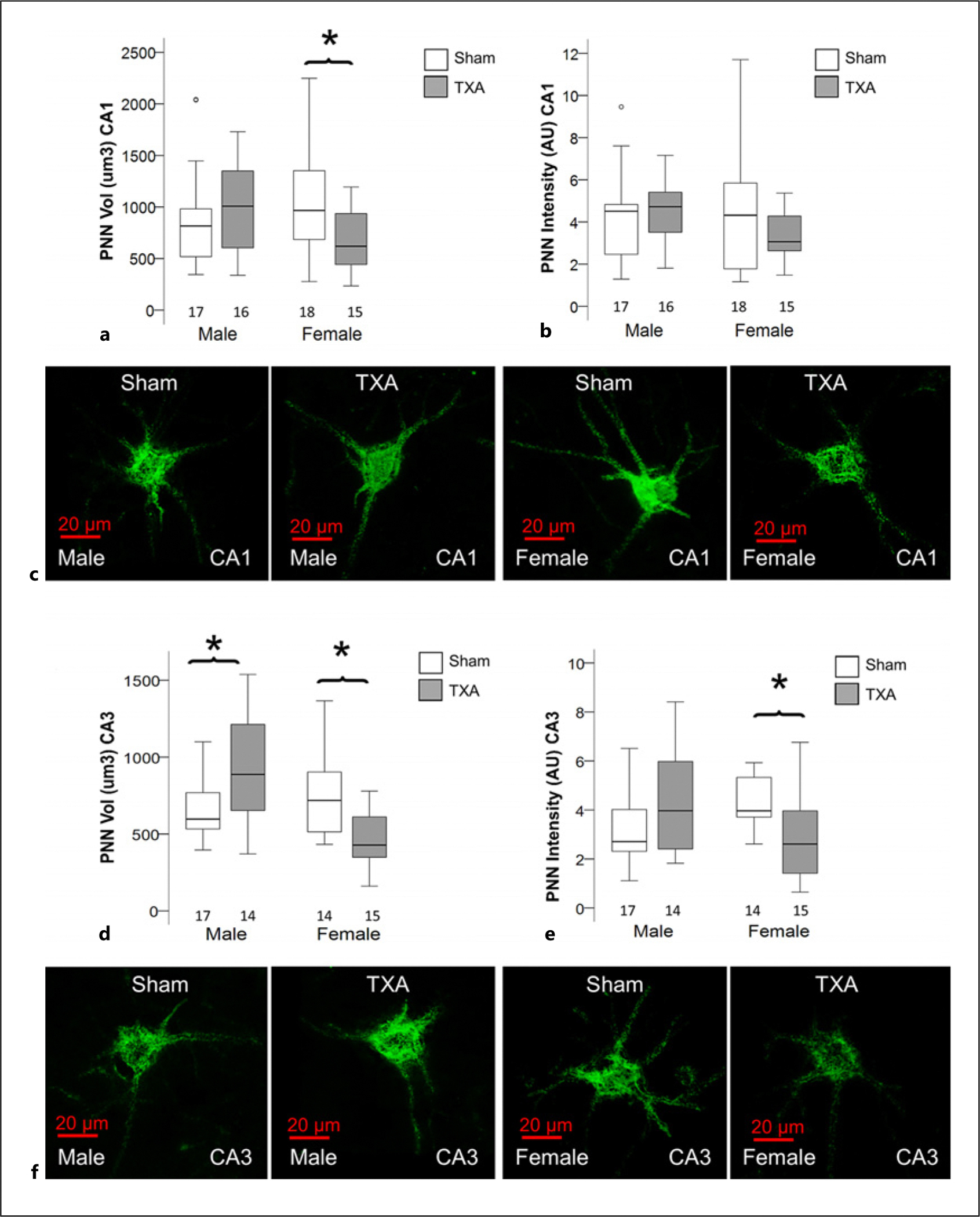
Disturbed PNN formation in the dorsal hippocampus of female IUGR mice at P60. Analysis of z-stacks captured from fluorescein-conjugated WFA-stained PNN in green channel in the dorsal hippocampus. Box and whiskers plots represent (**a, d**) total volume (in μm^3^ × 10^3^ μm^3^) and (**b, e**) total WFA IF (AU) at P40 in CA1 and CA3, respectively, stratified by sex. The number of PNN measured per group is depicted in each panel. Boxes are limited by the 75th and 25th percentiles (interquartile range [IQR]), and whiskers are limited by the last data point within 1.5 times the IQR from the median (continuous line inside the box). Mann-Whitney U test was applied. *p < 0.05. Unbiased image processing and analysis was performed using Imaris x64 v9.8 software blinded to treatments and sex. Representative surface reconstruction renderings from z-stacks using Zen blue software in CA1 (**c**) and CA3 (**f**), showing reconstructed PNNs (green channel). AU arbitrary units; CA, cornus ammonis; PNN, perineural nets; TXA, thromboxane A_2_-analog; WFA, Wisteria floribunda lectin; IF, immunofluorescent.

PNN formation is one of the key events closing the period of synaptic plasticity and stabilizing synaptic networks. Disrupted PNN formation occurred predominantly in IUGR female mice at P40 [19]. At P60, changes in PNN formation were also sexually dimorphic, with 31% (MW U (116.5) *p* = 0.036 vs. sham) and 42% (MW U (40.0) *p* = 0.004 vs. sham) smaller PNN volumes in CA1 (Fig. 3a, c) and CA3 (Fig. 3d, f) of IUGR female mice. Additionally, a 45% decrease (MW U (51.5) *p* = 0.018 vs. sham) in PNN intensity was observed in CA3 of IUGR female mice (Fig. 3e). In contrast to IUGR female mice, PNN volume in the CA3 of IUGR male mice was 42% larger (MW U (171.5) *p* = 0.036 vs. sham; Fig. 3d).

**Fig. 4.**
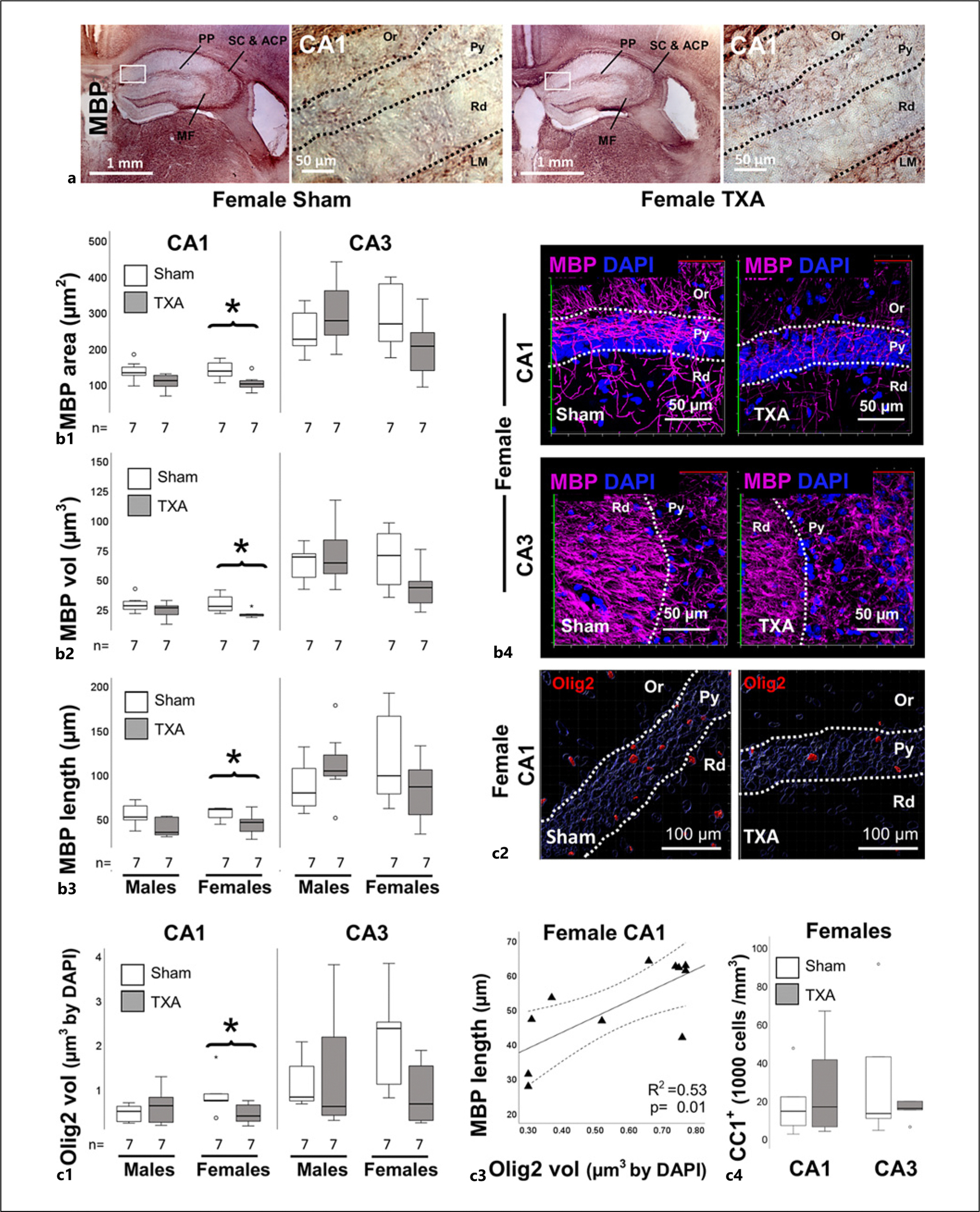
Hippocampal hypomyelination and decreased Olig2 expression in female IUGR mice at P60. **a** Representative images demonstrate major myelinated white matter tracks in the mossy fibers (MFs), Schaffer collaterals (SC), anterior commissural path (ACP), and the perforant path (PP) using MBP IHC. **b1–b3** Analysis performed in z-stacks captured from immunolabeling detecting MBP IR in magenta (Alexa 647 goat anti-mouse) and DAPI nuclear staining in the dorsal hippocampus. Box and whiskers plots represent filament analysis using Imaris software for MBP-stained myelinated axons in the CA1 and CA3, and stratification by sex. Analysis included total filament surface area (μm^2^, **b1**), volume (μm^3^, **b2**), and length (μm, **b3**). Representative renderings of z-stacks produced using Zen blue software for CA1 and CA3 (**b4**) sham and IUGR (TXA) female mice are shown. (**c1**) Olig2 volume (adjusted by number of nuclei/DAPI) is shown for CA1 and CA3 and (**c2**) representative Imaris 3D renderings for CA1 are shown. (**c3**) MBP^+^ filament length (μm; *y*-axis) directly correlated with Olig2 volume (μm^3^; *x*-axis) at P60 in the female mice. Continuous line represents the fitted line derived from a linear regression and the discontinuous line represents the 95% CI. (**c4**) The number of APC/CC1^+^ mature oligodendrocytes in 1,000 per mm^3^ is represented. Boxes are limited by the 75th and 25th percentiles (interquartile range [IQR]), and whiskers are limited by the last data point within 1.5 times the IQR from the median (continuous line inside the box), with outliers represented as °. Mann-Whitney U test was applied. *p < 0.05. Unbiased image processing and analysis was performed using Imaris x64 v9.8 software blinded to treatments and sex. CA, cornus ammonis; Or, oriens layer; MBP, myelin basic protein; Py, pyramidal cell layer; R^2^, coefficient of determination; Rd, radiatum layer.

**Fig. 5.**
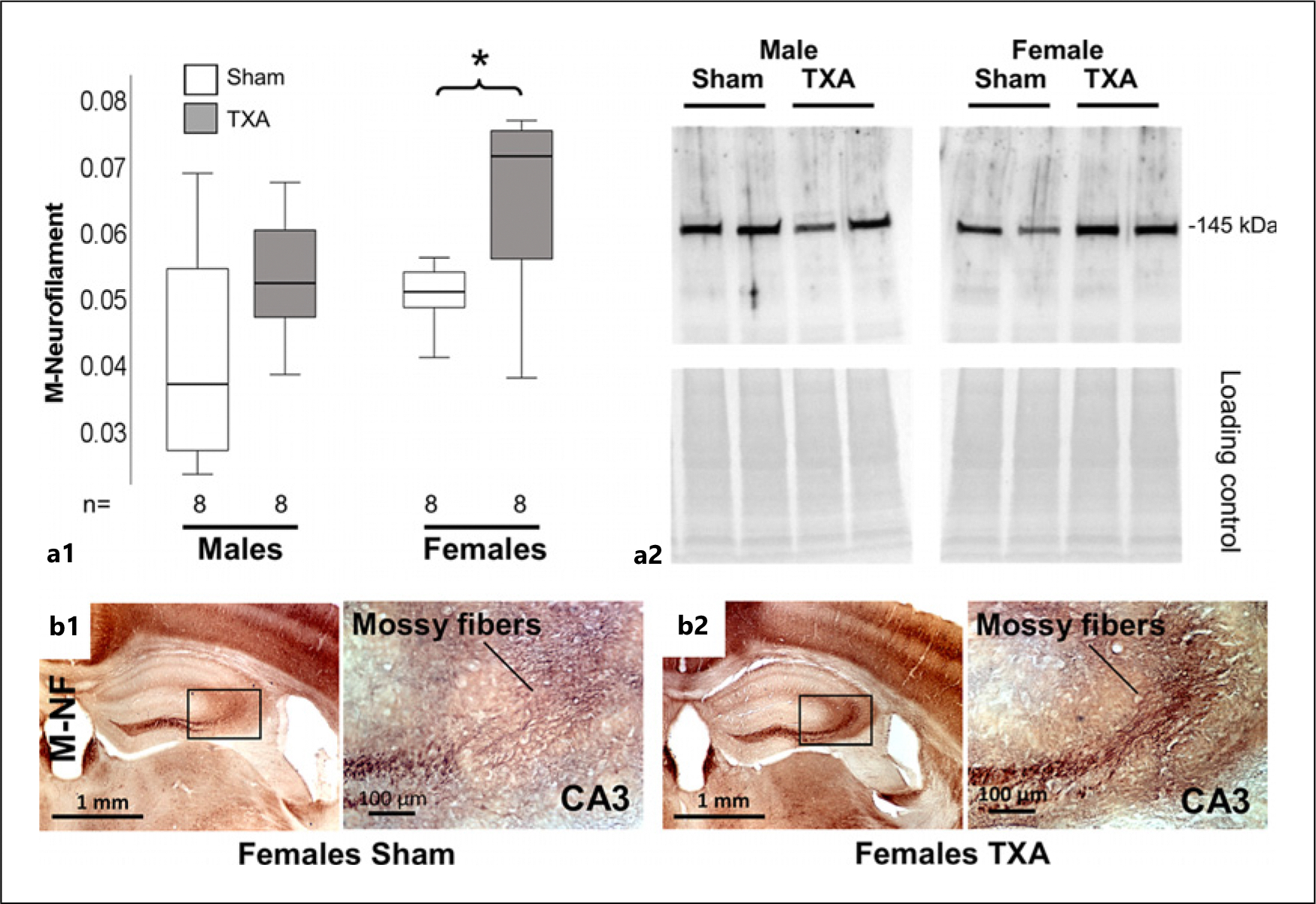
Increased M-neurofilament expression in the MF of female IUGR mice at P60. **a1** Expression of medium chain (M) neurofilament in hippocampal crudes relative to loading/transfer control in Western blot experiment. Boxes are limited by the 75th and 25th percentiles (interquartile range [IQR]), and whiskers are limited by the last data point within 1.5 times the IQR from the median (continuous line inside the box), with outliers represented as °. Mann-Whitney U test was applied. *p < 0.05. **a2** Representative Western blot is shown with single band at 145 kDa. **b1, b2** In immunohistochemistry experiment, M-NF was found overexpressed particularly in the main bundle of the MF as shown in images depicting sham and TXA female hippocampus. TXA, thromboxane A_2_-analog.

Myelination is another important event closing the pCP of synaptic plasticity in the hippocampus. Initially, we detected no differences in white matter tracks including the mossy fibers (MFs), Schaffer collaterals, anterior commissural path, and the perforant path using MBP IHC (Fig. 4a). When we evaluated these white matter tracks using 3D reconstruction techniques and Imaris filament analysis, we found that while no deficits were detected in hippocampal myelination in male IUGR mice at P60, the area (μm^2^), volume (μm^3^), and length (μm) of myelinated axons (MBP^+^) were decreased in the CA1 of IUGR female mice at P60 (all *p* < 0.05 vs. sham, *n* = 7 per group; Fig. 4b1–b3). Despite not reaching statistical significance, we noted a trend toward deficits in the CA3 of IUGR female mice as well. Representative 3D renderings for the CA1 of female mice are shown (without Imaris reconstruction) in Figure 4b4. No differences in myelination were detected in the hippocampal DG (data not shown). Next, we studied if differences in myelination correlated with deficits in oligodendrocyte abundance or maturation using the nuclear marker Olig2. The number of Olig2^+^ nuclei was similar between sham and IUGR mice of either sex, when adjusted either to the number of DAPI-stained nuclei or the total volume of the z-stack (data not shown). However, the abundance of Olig2 expression (volume of nuclear staining), a transcriptional factor directly linked to the myelination process in oligodendrocytes [50], was decreased in the CA1 of IUGR versus sham female mice (MW U (4.0) *p* = 0.045, *n* = 7/group) with a similar trend in CA3 (MW U (5.0) *p* = 0.068, Fig. 4c1). Representative Imaris reconstructions of Olig2 renderings for female CA1 sham and IUGR mice are shown in Figure 4c2. The abundance in the nuclear (DAPI co-labeled) Olig2 directly correlated with the length of MBP^+^ axons in the CA1 of female mice (Spearman Rho 0.603, *p* = 0.01, *R*^2^: 0.53; Fig. 4c3). Because the number of mature myelinating APC/CC1^+^ oligodendrocytes was similar between treatment groups at either subfield in female mice (Fig. 4c4), we speculate that MBP deficit in IUGR female mice was likely linked to oligodendrocytes dysmaturation/dysfunction. Similar direct correlations were found between Olig2 and MBP area (Rho: 0.822, *p* = 0.002) as well as volume (Rho: 0.758, *p* = 0.007). No differences in Olig2 were identified in the DG of either male or female IUGR mice (data not shown). Deficits in MBP and Olig2 contrast with the increase in M-NF by 38.5% in the hippocampus of IUGR female mice (MW U (53) *p* = 0.028 vs. sham, *n* = 8/group; Fig. 5a), which appears to be robustly expressed in the MFs (Fig. 5b), which connect DG to CA3. We speculate that impaired myelination at earlier time points (i.e., at P40) as shown previously [19] resulted in disrupted closure of the pCP of synaptic plasticity in the hippocampus of IUGR mice, particularly in females, which in turn allows for axonal outgrowth.

### Morphometric Analysis of Astrocytes and Microglia Support an “Active” State Not Linked to a Pro-Inflammatory Profile in Female IUGR Offspring during Early Adulthood

**Fig. 6.**
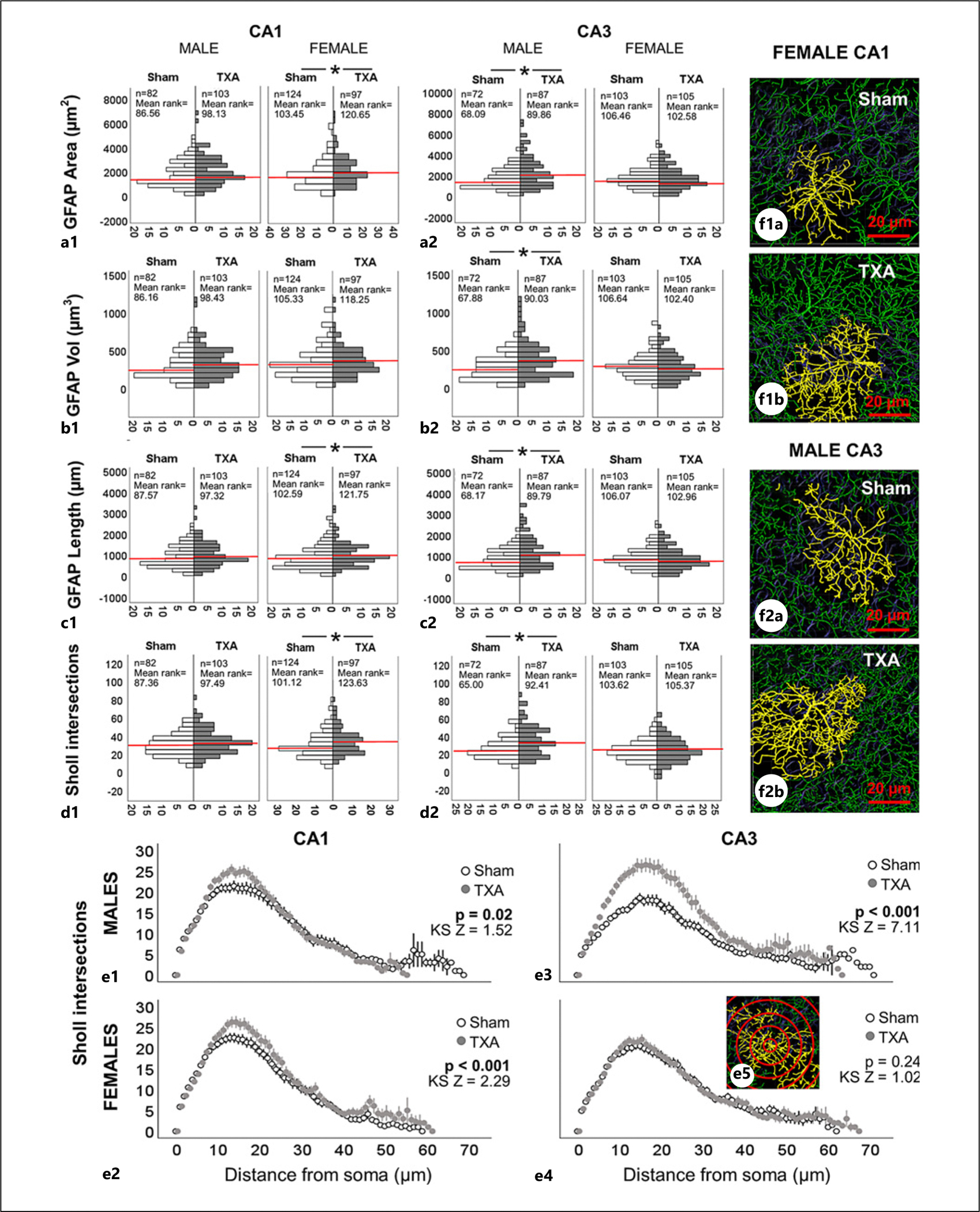
Morphometric analysis of astrocytes supports sexual dimorphism in the CA1 and CA3 subfields of IUGR mice at P60. Analysis performed in z-stacks captured from immunolabeling detecting GFAP IR in green (Alexa 488 goat anti-chicken) and DAPI nuclear staining in dorsal CA1 and CA3. **a–d** Bar graph represents frequency distribution of all GFAP-expressing astrocytes for (**a**) surface area (μm^2^), (**b**) volume (μm^3^), and (**c**) length of astrocytic processes (μm) as well as (**d**) total number of Sholl intersections for sham (white bars) and TXA (gray bars) CA1 and CA3 by sex. Red line indicates median. Sample size (n) and mean rank are provided as well. Mann-Whitney U test was applied. *p value <0.05. **e** Distribution of Sholl intersections (*y-axis*) away from the soma (in μm, *x-axis*) is shown along with Kolmogorov-Smirnov (KS) statistic Z and *p* value to determine difference in distribution. **f** Representative Imaris 3D renderings for female CA1 and male CA3 are shown. Unbiased image processing and analysis was performed using Imaris x64 v9.8 software blinded to treatments and sex. CA, cornus ammonis; GFAP, glial fibrillary acid protein; S, sham; TXA, thromboxane A_2_-analog.

Astrocytes and microglia play a role in the progression of the pCP of ocular dominance [51–53] and auditory development [54], but their role in modulating synaptic plasticity in the hippocampus is less known. Thus, we studied if sexual dimorphism in astrocytic and microglial morphometric evidence of activation at P60 existed to shed light on a mechanistic role in the disruption of the pCP of synaptic plasticity in the IUGR hippocampus. In this model, the number of astrocytes (GFAP^+^) and microglia/infiltrating macrophages (Iba1^+^) was similar in the sham and IUGR CA1 and CA3 subfields of both sexes (data not shown). However, sexual dimorphism in morphology of both astrocytes and microglia was identified between sham and IUGR mice. In the CA1, astrocytes from female IUGR mice had larger surface area (11.4%, MW U [6,950.5] *p* = 0.04, Fig. 6a1), greater total length of projections (11.9%, MW U [7,057.0] *p* = 0.02, Fig. 6c1), and higher number of Sholl intersections (22.2%, MW U [7,239.5] *p* = 0.009, Fig. 6d1) than those of female sham mice. Conversely, astrocytes in the CA3 of male IUGR mice had larger surface area (52.8%, MW U [3,989.5] *p* = 0.003, Fig. 6a2), volume (54.5%, MW U [4,004.5] *p* = 0.003, Fig. 6b2), length of projections (47%, MW U [3,983.5] *p* = 0.003, Fig. 6c2), and Sholl intersections (36.2%, MW U [4,212.0] *p* < 0.001, Fig. 6d2). Astrocytic branching was more robust in the CA3 of male IUGR mice (*p* < 0.001, Fig. 6e2), but significant differences are also documented in the CA1 of both male (Fig. 6e1) and female (Fig. 6e3) IUGR mice. Representative 3D rendering of isolated astrocytes is shown in yellow for IUGR female CA1 (Fig. 6f1) and IUGR male CA3 (Fig. 6f2). Despite the increased astrocytic branching and volume in the CA3 of IUGR males, no domain overlapping was detected, which along with a lack of increased numbers suggests an alteration in astrocytic function not likely linked to reactive astrogliosis as seen in other models [44, 48].

**Fig. 7.**
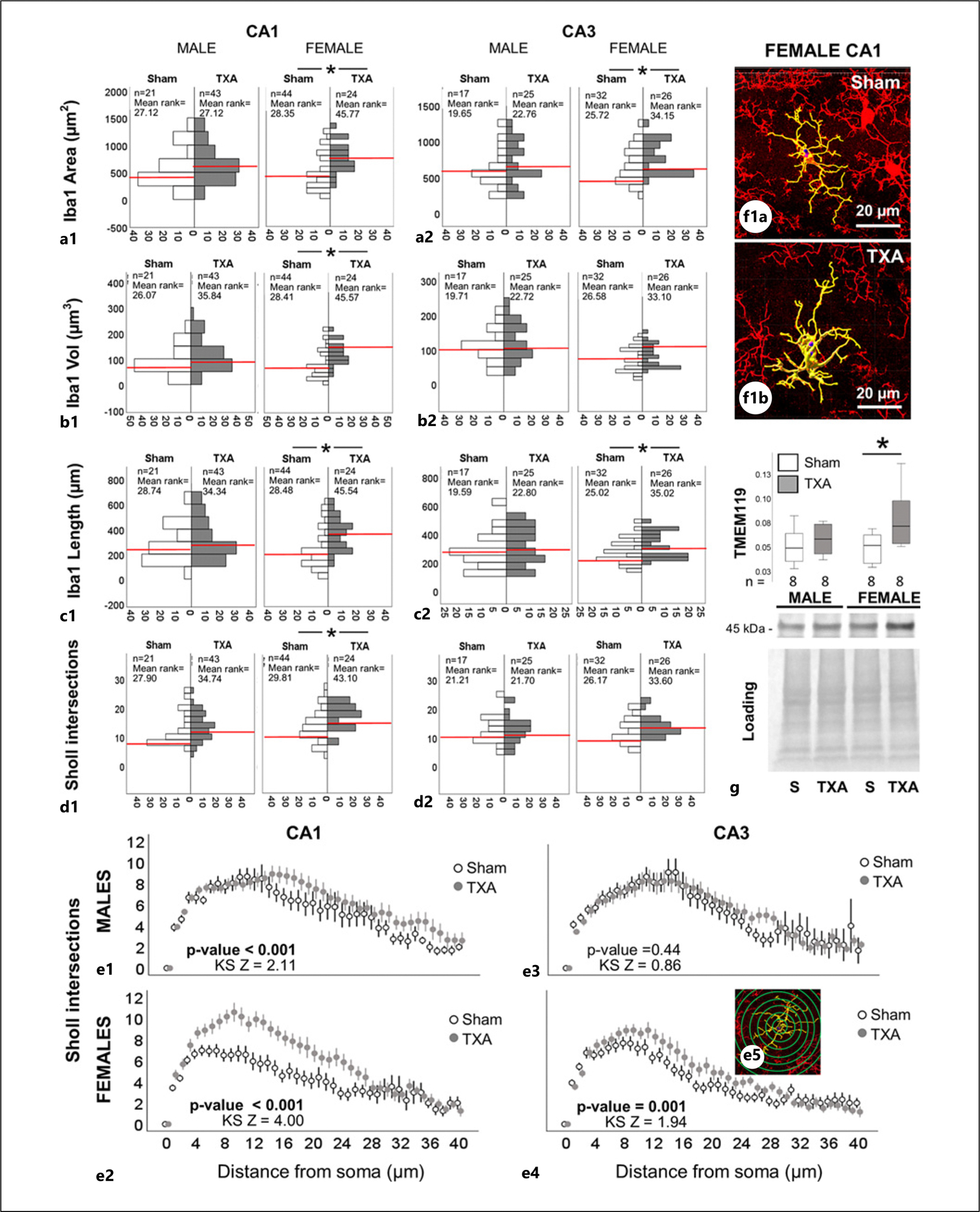
Morphometric analysis of iba ^+^ cells supports hyper-ramified microglia in the CA1 and CA3 subfields of female IUGR mice at P60. Analysis performed in z-stacks captured from immunolabeling detecting iba1 IR in red (Alexa 568 goat anti-rabbit) and DAPI nuclear staining in dorsal CA1 and CA3. **a–d** Bar graph represents frequency distribution of all Iba1-expressing cells for (**a**) surface area (μm^2^), (**b**) volume (μm^3^), and (**c**) length of processes (μm) as well as (**d**) total number of Sholl intersections for sham (white bars) and TXA (gray bars) CA1 and CA3 by sex. Red line indicates median. Sample size (n) and mean rank are provided. Mann-Whitney U test was applied. *p value <0.05. **e** Distribution of Sholl intersections (*y-axis*) away from the soma (in μm, *x-axis*) is shown along with Kolmogorov-Smirnov (KS) statistic Z and *p* value to determine difference in distribution. **f** Representative Imaris 3D renderings for female CA1 are shown. Unbiased image processing and analysis was performed using Imaris x64 v9.8 software blinded to treatments and sex. **g** Expression of TMEM119 in hippocampal crudes relative to loading/transfer control in Western blot experiment. Boxes are limited by the 75th and 25th percentiles (interquartile range [IQR]), and whiskers are limited by the last data point within 1.5 times the IQR from the median (continuous line inside the box), with outliers represented as °. Mann-Whitney U test was applied. *p < 0.05. Representative Western blot is shown with single band at 45 kDa. CA, cornus ammonis; Iba1, Ionized calcium binding adapter molecule 1; S, sham; TMEM119, transmembrane protein 119; TXA, thromboxane A_2_-analog.

Unlike astrocytes, morphometric analysis of Iba1^+^ cells denoting microglia and infiltrating macrophages supported an “active” state in both CA1 and CA3 subfields only in IUGR female mice. As such, Iba1^+^ cells in the CA1 of IUGR females had larger areas (MW U (789.5) *p* < 0.001, Fig. 7a1), volumes (MW U [796] *p* < 0.001, Fig. 7b1), length of projections (MW U [793] (*p* < 0.001, Fig. 7c1), and number of Sholl intersections (MW U [734.5] *p* = 0.008, Fig. 7d1) versus sham female. Similarly, Iba1^+^ cells in the CA3 of IUGR female mice were larger in area (MW U [537] *p* = 0.05, Fig. 7a2) and length of projections (MW U [559.5] *p* = 0.025, Fig. 7c2) versus sham female. Distribution of Sholl intersections demonstrated the most robust branching around 6–12 μm from the soma in the CA1 Iba1^+^ cells of IUGR female mice (*p* < 0.001, Fig. 7e3) compared to sham at 4 μm from the soma, with representative panels of isolated microglia shown in Figure 7f1. To differentiate changes in microglia versus infiltrating macrophages, we use the specific microglia marker TMEM119 in hippocampal homogenates. TMEM119 was increased by 23.6% only in the IUGR female hippocampus in immunoblots (MW U [49] *p* = 0.02 vs. sham, Fig. 7g), suggesting that microglia represent the most abundant cell type in the Iba1^+^ population. Next, we tested whether these morphometric differences in microglia, which supported an “active” hyper-ramified bushy state, resulted in prolonged neuroinflammation into early adulthood. Using the highly sensitive Meso Scale Discovery platform, we did not detect differences between groups (online suppl. Table), suggesting that these “active” microglia serve a function distinct from inflammation.

## Discussion

**Fig. 8.**
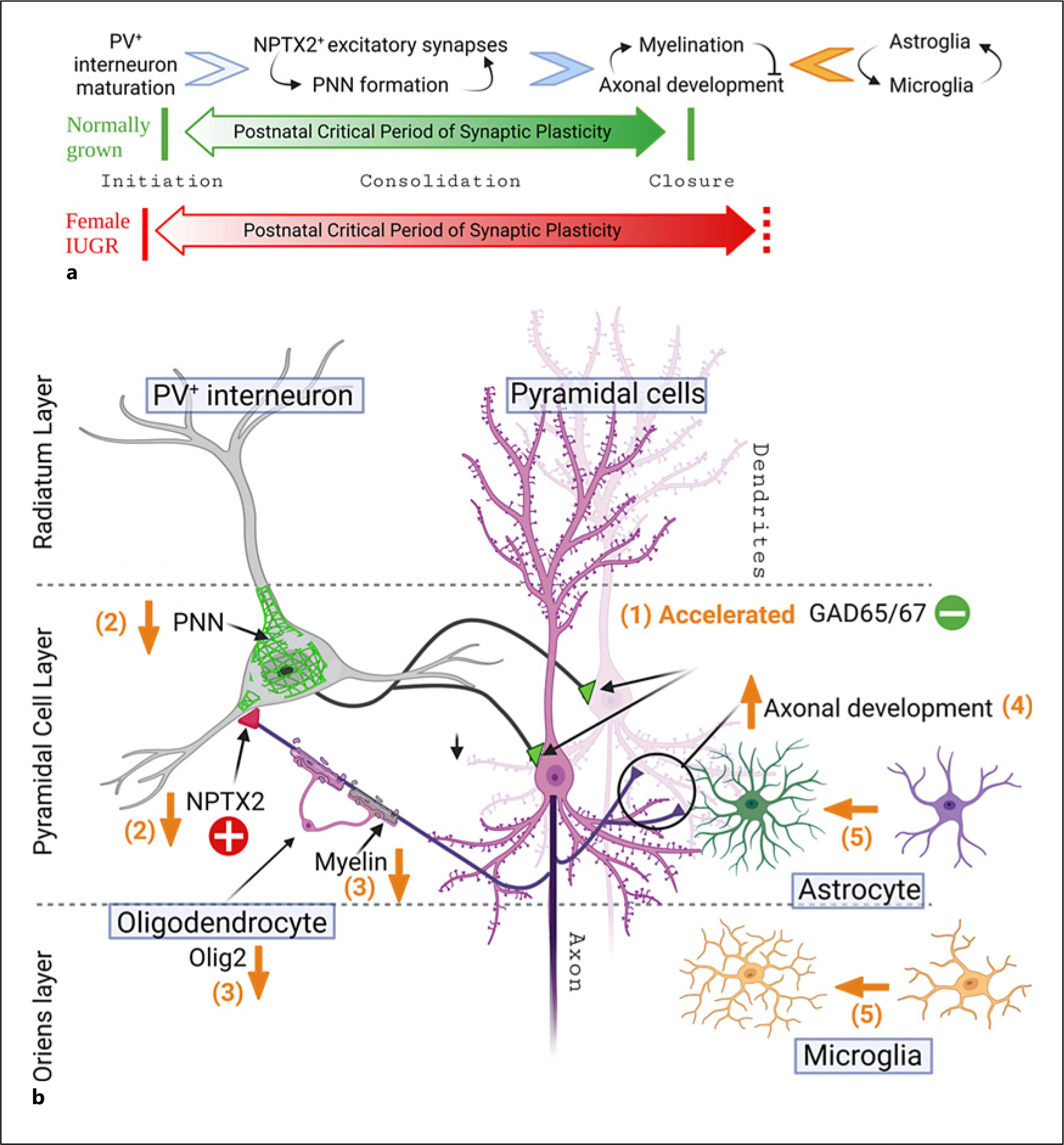
IUGR conceptual framework supporting disruption of events governing the boundaries of the pCP in the hippocampus. **a** Maturation of PV^+^ interneurons is one of the most important events triggering the onset of the pCP, leading to increased expression and function of NPTX2, which in turn stabilizes AMPA receptors modulating excitatory inputs onto PV^+^ interneurons supported by PNN formation to establish the E/I balance during the process of consolidation and closure of the pCP [18, 58]. The decrease in GAP43, NogoR1, Nogo-A, MAG, and OMG that follows is known to inhibit neurite outgrowth and axonal development, allowing the progression of myelination [27–29]. Astrocytes and microglia play a role in the progression of the pCP of ocular dominance [51–53] and auditory development [54], but their role in modulating synaptic plasticity in the hippocampus is less clear, likely focused on synaptic maintenance and pruning in response to synaptic development [56, 57]. **b** Based on the work presented here and previously [19], we proposed the following conceptual framework supporting the disruption of the pCP thesis, including: (1) accelerated GABAergic maturation resulting in increased GAD 65/67^+^ synaptic buttons perisomatic to hippocampal pyramidal cell; (2) premature inhibition of pyramidal cells prevents NPTX2 transcription and stabilization of AMPA receptors on PV^+^ interneurons, which as a result prevents the normal developments of PNN; (3) downstream from these deficits impaired myelination follows likely due to oligodendroglia dysfunction; (4) decreased myelination allows neurite outgrow and axonal development; and (5) decreased myleination results in increased morphometric complexity of both the astrocytes and the microglia, likely due to increased pruning. GAP, growth-associated protein; MAG, myelin-associated glycoprotein; NOGO, neurite outgrowth inhibitor; NPTX2, neuronal pentraxin 2; OMG, oligodendrocyte-myelin glycoprotein; pCP, postnatal critical period of synaptic plasticity; PNN, perineural nets; PV, parvalbumin; E/I, excitatory/inhibitory.

Here, we report for the first time how HDP-induced IUGR disrupts the progression of events governing the closure of the pCP in the hippocampus up to early adulthood in a sexually dimorphic manner (Fig. 8). We speculate that the increased vulnerability to worse memory performance in IUGR females reported in our model [17] may be in part the result of this disruption, which mechanistically stems from a differential susceptibility to impair myelination, promoting axonal overgrowth and likely the morphometric microglia changes. Although IUGR offspring from both sexes develop hippocampal hypoplasia as late as P60, greater disruption of the pCP occurs in IUGR females up to early adulthood, a time at which this period should have been closed in the rodent hippocampus [23, 32–35]. Specifically, in adult IUGR females, deficits in NPTX2^+^ excitatory puncta coincide with disruption in PNN formation, while impaired myelination corresponds to deficits in nuclear Olig2 expression in oligodendrocytes. The importance of myelination in closing the pCP has been extensively reported [19, 29, 33–35]; thus, decreased expression of nuclear Olig2 with preserved number of Olig2 and APC/CC1-expressing oligodendrocytes may suggest oligodendrocyte dysfunction in IUGR female mice explained decreased myelination [50]. Impaired timely closure of the pCP permits axonal and synaptic overdevelopment which may underlie the increase in M-NF, particularly in the MFs of these IUGR female mice. Both astrocytes and microglia play critical roles in neuroinflammation [55] and synaptic pruning in response to excessive synaptic development [56, 57]. Our morphometric analysis demonstrates sexual dimorphism with increased astrocytic size and complexity in both sexes but increased microglia hyper-ramification only in IUGR females, which are not associated with a pro-inflammatory cytokine milieu. Thus, we speculate that one potential mechanism supporting sexual dimorphism in our IUGR model may include a greater susceptibility of female oligodendrocyte to dysfunction after IUGR, impairing myelination, allowing axonal overgrowth, and leading to increased microglia-mediated synaptic pruning but not necessarily ongoing neuroinflammation (Fig. 8b). The link of the events described here with the learning and memory deficits observed in our adult female IUGR mice [17] and the cognitive disorders seen in human IUGR offspring [8, 9] requires further examination.

Sex differences in the effects of IUGR have been reported in humans. Human females have an advantage over males in the setting of fetal undernutrition or maternal malnutrition, resulting in early onset IUGR, as female fetuses survive in a greater proportion [59–61] with epigenetic changes supporting this protection [62]. No sex differences are reported in early outcomes of live IUGR human offspring (i.e., rate of prematurity, intraventricular hemorrhage, or respiratory distress syndrome) [63]. However, in the setting of prematurity (24–29 weeks of GA), male infants born with history of early onset IUGR have worse school achievement and behavioral problems than female premature infants [64, 65], while female infants regardless of gestational age suffering from severe IUGR (early and late onset) have worse neurocognitive impairment than male infants [66]. Thus, it is important to acknowledge that males and females vary in their susceptibilities to different neurobehavioral deficits based on the onset of the pathology leading to IUGR. In our experiments, we have modeled the effect of UPI as seen in HDP-inducing IUGR during the second half of human pregnancy, which results in worse neurocognitive impairment in female than male infants [66].

In our model, all sham and IUGR pups are cross-fostered to unmanipulated mouse dams made pregnant at the same time as the dams destined for sham or IUGR surgery to avoid any lactation issues associated with anesthesia and micro-osmotic pump implantation. Thus, we assume that sham and IUGR pups receive similar amounts of dam milk during postnatal rearing, with which our IUGR females achieve catch-up growth 7 weeks later than IUGR males (11 weeks vs. 4 weeks) [15]. There is ample literature to show that attaining catch-up growth is paramount to determining future neurologic health. Adequate catch-up reduces learning and memory impairment among other neuropsychological detriments [67–69]. The lack of catch-up growth in IUGR females could incur an additional postnatal growth restriction which may impact brain growth and function. At this juncture, the reasons behind why IUGR females fail to attain catch-up growth as compared to IUGR males are unknown. We believe that one contributor may relate to the sexually dimorphic placental nutrient transporter expression found in late gestation in this model. Placenta of IUGR males upregulated neutral amino acid transporters as well as fatty acid transporters as compensatory mechanisms to improve fetal nutrient accretion [70]. Thus, IUGR males may also be at increased risk for earlier onset metabolic syndrome with faster catch-up growth, supported by their heavier BWs at 8.5 months compared to sham males [15]. The competing interests of attaining catch-up growth to improve neurocognitive and neuropsychiatric outcomes but to avoid worse metabolic outcomes remain a clinical challenge in the care of IUGR infants.

After correction for sex, human IUGR offspring have smaller volumes of limbic structures [71], including the hippocampus, supporting the known cognitive and memory deficits [4, 6, 11, 13, 72]. These hippocampal hypoplasia and memory deficits were replicated by us in the HDP-induced IUGR mouse model up to P40 (equivalent to human adolescence) [17] and persist to P60 in both sexes, as shown in this current study. We find that hippocampal hypoplasia is not explained by overall cerebral hypoplasia in IUGR mice, and thus, like in humans [71, 73, 74], IUGR appears to produce a regional and not a global injury. Despite no differences in hippocampal hypoplasia, we identified persistent deficits in the events governing the closure of the pCP, particularly affecting IUGR female mice.

We have previously reported that following accelerated GABAergic development, NPTX2 ^+^ puncta (from excitatory synapses) are persistently decreased, a deficit that correlates with impaired myelination and PNN integrity preferentially in female IUGR mice up to P40 [19]. Here, we report that NPTX2 deficits persist in IUGR female mice as late as P60. Because NPTX2 is produced by excitatory neurons and accumulates in excitatory synapses targeting PV-expressing interneurons [26], mediating activity-dependent plasticity [75] and memory formation [31, 76–81], we speculate that accelerated GABAergic maturation [19] in fact negatively impacted pyramidal cell activity, leading to persistent NPTX2 downregulation. Because NPTX2 is paramount in enhancing and maintaining PNN formation [82], the decrease in PNN volume and intensity in IUGR female mice is likely a downstream effect. Further evidence of disruption of the events governing the closure of the pCP in the dorsal hippocampus includes impaired myelination and decreased Olig2 expression in oligodendrocytes. Deficits in oligodendrocytes and myelination are reported at different time points in our HDP-mediated IUGR mouse model [10, 19], as well as in a sheep [83], and other IUGR models [42, 55, 84]. Here, we did not find a decreased number of oligodendrocytes in the hippocampus of the IUGR offspring of either sex at P60; instead, we documented a decreased nuclear expression of Olig2, a known marker of oligodendrocytes at all stages of development and a transcription factor needed for maturation [50]. The direct correlation between Olig2 nuclear expression and MBP length and the lack of differences in the number of APC/CC1^+^ mature oligodendrocytes support the functional effect in myelination and the deficits seen only in IUGR female offspring.

One of the broad consequences of disrupting the timely closure of the pCP of synaptic plasticity in the hippocampus is the deterrence of the consolidation of downstream pCPs in other brain regions, which depend on mature hippocampal networks for acquisition of higher brain functions and networks [22–24, 34]. Delayed or stalled myelination, which inhibits axonal growth [85], results in axonal overgrowth. The increase in M-NF in female IUGR hippocampus at P60 provides support for this notion. The end result of redundancy in synaptic networks will require careful assessment in future mechanistic and neurobehavioral experiments during aging, knowing that sex dimorphism exists in most neurodevelopmental, neuropsychiatric, and neurodegenerative disorders [86, 87].

We remain intrigued about the implications of increased hyper-ramified bushy microglia at P60 in the hippocampal CA subfields of the IUGR female mice. Despite finding no differences in the hippocampal cytokine/chemokine milieu between sham and IUGR of either sex at P60, hyper-ramified bushy microglia could play an essential role in activity-dependent refinement (synaptic pruning) of neuronal networks during the pCP of synaptic plasticity [88, 89]. We are cognizant that minimal changes in microglia function can lead to significant alterations in brain development, as these cells are exquisitely sensitive to their environment [86]. Thus, our interpretation of the morphometric changes observed in microglia from IUGR female mice remains that of a response to axonal overgrowth and excessive synaptic development. Ongoing studies are underway to dissect the final synaptic composition in the IUGR hippocampi by examining pre- and postsynaptic densities. Lastly, a second alternate hypothesis may involve functional microglial changes secondary to sex hormone derangements originating from IUGR [90–93], aspects that are beyond the scope of this current study but are certainly important contributors to understanding the sexual dimorphism of IUGR-induced brain injury.

We acknowledge certain limitations to this study. First, the mechanistic aspects of how these events occur and the downstream outcomes are still under investigation. Studies of many other events important in governing the pCP in the hippocampus such as neurotrophic support are needed. The link between sexually dimorphic microglia morphology and closure of the pCP needs further study, as mentioned before. Lastly, future studies incorporating the functional integration of the hippocampus with other structures such as the entorhinal cortex in response to IUGR are warranted.

## Conclusions

This study is a continuation of our previous studies [17, 19], reporting on the premature initiation in the pCP in the dorsal hippocampus linked to accelerated postnatal GABAergic development in IUGR offspring from both sexes. Here, we extended the longitudinal examination of events critical to the closure of the pCP by interrogating NPTX2 expression, PNN formation, and axonal myelination, all of which are disrupted more prominently in IUGR female mice, suggesting that the closure is delayed in female offspring up to early adulthood. The role of microglia existing in an “active” state as the cause or the result of these dysregulated events needs further investigation (Fig. 8b). Our body of work supports the thesis that premature onset coupled with prolonged lengthening and closure of the pCP in the dorsal hippocampus may be one mechanism that may underlie learning and memory deficits in postnatal life of IUGR offspring. Therapies targeted at preventing postnatal accelerated GABAergic development and supporting myelination may reframe the boundaries of the pCP and may prevent the neurodevelopmental and neuropsychiatric disorders well reported in human offspring [94].

## Supplementary Material

Suppl Table 1

Suppl Fig 1

## Data Availability

All data generated or analyzed during this study are included in this article and its supplementary material files. Further inquiries can be directed to the corresponding author. Supplementary material is available online at www.karger.com/doi/10.1159/000530451.
