# Supplementary material for: Sexual Dimorphism in the Closure of the Hippocampal Postnatal Critical Period of Synaptic Plasticity after Intrauterine Growth Restriction: Link to Oligodendrocyte and Glial Dysregulation
Source: Dev Neurosci. Author manuscript; Available in PMC 2025 May 26. (PMC12105909; doi:10.1159/000530451)
Supplement: Suppl Table 1 [file NIHMS2075725-supplement-Suppl_Table_1.docx]

**Supplemental table: Cytokine/ chemokine profile in P60 hippocampus**

| **Table A** | **Rx** | **Percentiles** | | |  |  |
| --- | --- | --- | --- | --- | --- | --- |
| **ALL** |  | **25** | **50** | **75** | **n** | **p-value** |
| **IFNγ** | Sham | .00000 | .01000 | .01000 | 15 | 0.285 |
|  | TXA | .00750 | .01000 | .01250 | 10 |  |
| **IL1-β** | Sham | .2200 | .2800 | .3400 | 15 | 0.599 |
|  | TXA | .2100 | .3250 | .3325 | 10 |  |
| **IL-5** | Sham | .0600 | .0700 | .0800 | 15 | 0.873 |
|  | TXA | .0500 | .0650 | .0825 | 10 |  |
| **IL-6** | Sham | 1.0600 | 1.2000 | 1.6400 | 15 | 0.664 |
|  | TXA | 1.0425 | 1.3300 | 1.5525 | 10 |  |
| **CXCL1** | Sham | 5.7500 | 6.3200 | 8.9500 | 15 | 0.664 |
|  | TXA | 6.2775 | 7.1750 | 9.1350 | 10 |  |
| **TNFα** | Sham | .0700 | .0900 | .1200 | 15 | 0.397 |
|  | TXA | .0600 | .0850 | .1200 | 10 |  |

| **Table B** | **Rx** | **Percentiles** | | |  |  |
| --- | --- | --- | --- | --- | --- | --- |
| **MALES** |  | **25** | **50** | **75** | **n** | **p-value** |
| **IFNγ** | Sham | .00000 | .00500 | .01000 | 8 | 1.00 |
|  | TXA | .00000 | .01000 | .01000 | 5 |  |
| **IL-1β** | Sham | .2300 | .2950 | .3475 | 8 | 0.755 |
|  | TXA | .2000 | .3300 | .4050 | 5 |  |
| **IL-5** | Sham | .0450 | .0600 | .0700 | 8 | 0.573 |
|  | TXA | .0450 | .0700 | .0950 | 5 |  |
| **IL-6** | Sham | 1.1050 | 1.2300 | 1.3625 | 8 | 0.755 |
|  | TXA | 1.0650 | 1.2700 | 1.6750 | 5 |  |
| **CXCL1** | Sham | 5.4950 | 6.2700 | 8.5875 | 8 | 0.662 |
|  | TXA | 5.9000 | 6.8300 | 9.3250 | 5 |  |
| **TNFα** | Sham | .0475 | .0750 | .1125 | 8 | 0.573 |
|  | TXA | .0600 | .1100 | .1200 | 5 |  |

| **Table C** | **Rx** | **Percentiles** | | |  | **MWU** |
| --- | --- | --- | --- | --- | --- | --- |
| **FEMALES** |  | **25** | **50** | **75** | **n** | **p-value** |
| **IFNγ** | Sham | .00000 | .01000 | .01000 | 7 | 0.106 |
|  | TXA | .01000 | .01000 | .02000 | 5 |  |
| **IL-1β** | Sham | .2200 | .2600 | .3200 | 7 | 0.852 |
|  | TXA | .1900 | .2900 | .3250 | 5 |  |
| **IL-5** | Sham | .0600 | .0700 | .0900 | 7 | 0.662 |
|  | TXA | .0500 | .0600 | .0800 | 5 |  |
| **IL-6** | Sham | .9800 | 1.1100 | 1.7200 | 7 | 1.00 |
|  | TXA | .8650 | 1.3900 | 1.5450 | 5 |  |
| **CXCL1** | Sham | 5.9500 | 6.4300 | 10.6100 | 7 | 1.00 |
|  | TXA | 6.1850 | 7.5200 | 8.9750 | 5 |  |
| **TNFα** | Sham | .0900 | .1000 | .1600 | 7 | **0.043*** |
|  | TXA | .0650 | .0800 | .1100 | 5 |  |

*Values for IL-10, IL-12p70, IL-2, and IL-4 were below level of detection*

**IL**, interleukin; **INF**, interferon; **MWU**, Mann Whitney U test; **RX**, treatment group; **TNF**, tumor necrosis factor; **TXA**, thromboxane A_2_-analog (IUGR group).
