# Supplementary figures and images for: Sexual Dimorphism in the Closure of the Hippocampal Postnatal Critical Period of Synaptic Plasticity after Intrauterine Growth Restriction: Link to Oligodendrocyte and Glial Dysregulation
Source: Dev Neurosci. Author manuscript; Available in PMC 2025 May 26. (PMC12105909; doi:10.1159/000530451)

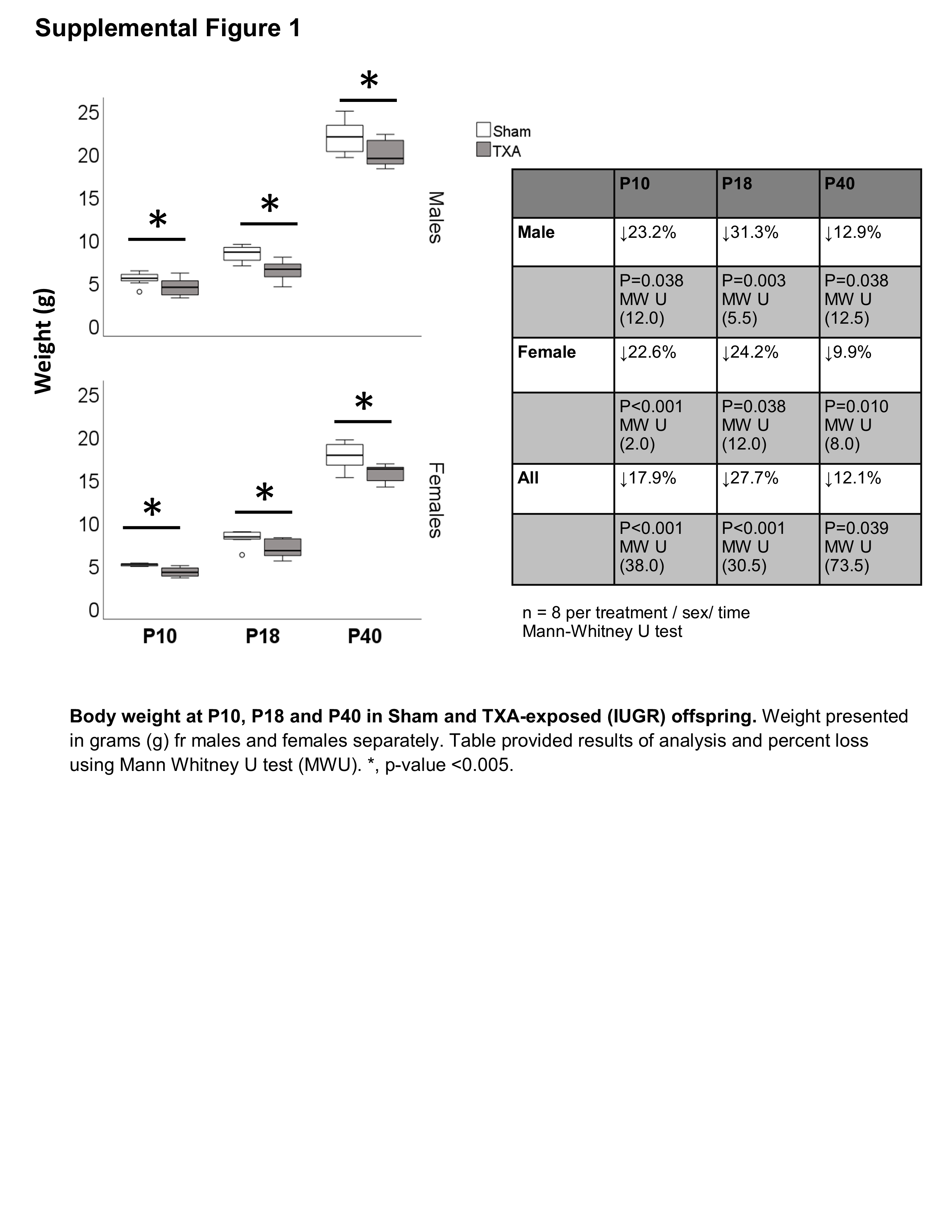

Supplement: Suppl Fig 1 [file NIHMS2075725-supplement-Suppl_Fig_1.tiff]
